# Large-scale Implementation of a COVID-19 Remote Patient Monitoring Program

**DOI:** 10.5811/westjem.60172

**Published:** 2023-10-27

**Authors:** Lulu Wang, Marisa Arky, Alyssa Ierardo, Anna Scanlin, Melissa Templeton, Ethan Booker

**Affiliations:** * MedStar Washington Hospital Center, Department of Emergency Medicine, Washington, DC; † MedStar Telehealth Innovation Center, MedStar Institute for Innovation, Washington, DC; ‡ Georgetown University Hospital and Washington Hospital Center Emergency Medicine Residency, Washington, DC

## Abstract

**Introduction:**

We implemented a large-scale remote patient monitoring (RPM) program for patients diagnosed with coronavirus 2019 (COVID-19) at a not-for-profit regional healthcare system. In this retrospective observational study, patients from nine emergency department (ED) sites were provided a pulse oximeter and enrolled onto a monitoring platform upon discharge.

**Methods:**

The RPM team captured oxygen saturation (SpO_2_), heart rate, temperature, and symptom progression data over a 16-day monitoring period, and the team engaged patients via video call, phone call, and chat within the platform. Abnormal vital signs were flagged by the RPM team, with escalation to in-person care and return to ED as appropriate. Our primary outcome was to describe study characteristics: patients enrolled in the COVID-19 RPM program; engagement metrics; and physiologic and symptomatic data trends. Our secondary outcomes were return-to-ED rate and subsequent readmission rate.

**Results:**

Between December 2020–August 2021, a total of 3,457 patients were referred, and 1,779 successfully transmitted at least one point of data. Patients on COVID-19 RPM were associated with a lower 30-day return-to-ED rate (6.2%) than those not on RPM (14.9%), with capture of higher acuity patients (47.7% of RPM 30-day returnees were subsequently hospitalized vs 34.8% of non-RPM returnees).

**Conclusion:**

Our program, one of the largest studies to date that captures both physiologic and symptomatic data, may inform others who look to implement a program of similar scope. We also share lessons learned regarding barriers and disparities in enrollment and discuss implications for RPM in other acute disease states.

Population Health Research Capsule What do we already know about this issue?

*Remote patient monitoring (RPM) is a versatile tool for management of patients with chronic disease states (eg, hypertension).*

What was the research question?

*Is a large-scale implementation of RPM feasible for patients with COVID-19? What are the associated barriers?*

What was the major finding of the study?

*With 3,457 patients enrolled in the COVID-19 RPM program, RPM was associated with a lower 30-day return-to-ED rate (6.2% vs 14.9% for controls).*

How does this improve population health?

*Remote patient monitoring is a lightweight and scalable tool to manage care for large populations with acute diseases states such as COVID-19.*

## INTRODUCTION

Coronavirus 2019 (COVID-19) created an urgent need for rapid adoption of telehealth. Hospital systems, faced with unprecedented demand on limited resources, needed a means to maximize inpatient capacity while minimizing infectious spread, and to redistribute care safely from the hospital to the community setting. Remote patient monitoring (RPM) offered one potential solution. Remote patient monitoring is the use of digital medical devices to collect and electronically transmit patient data from a remote site to drive care management.^1^ Frequently used devices include pulse oximeters, blood pressure cuffs, glucometers, and weight scales.^1^

Historically, RPM has been used to manage chronic diseases such as diabetes, hypertension, and chronic obstructive pulmonary disease,^2^ with demonstrated decrease in return-to-ED and hospital readmission rates.^3^ ^–^ ^5^ Due to its versatility, RPM emerged as a promising tool for COVID-19 management. It allows for timely detection of disease progression (as denoted by hypoxemia or tachycardia) and provides a venue for patients to report worsening symptoms. All together, these data points help clinicians identify when return to acute care is necessary.

Currently, little is known about the use of RPM in acute disease states for large populations. Baseline care inequity due to health disparities such as insurance status, English-speaking proficiency, and technologic fluency may be exacerbated in RPM. This study contributes knowledge on logistics for deploying a program that incorporates two often marginalized patient populations in RPM: patients with limited English proficiency, and those without smartphones. Furthermore, we share information regarding enrollment process, device supply and management, and staffing for a large-scale program deployed across a multiregional patient population. Our primary purpose in this retrospective observational study was to describe the methodology of deploying a COVID-19 RPM program at a multiregional hospital system and quantify its patient and program characteristics. We also share the return-to-ED rate and disposition of patients who return to acute care following COVID-19 RPM.

## METHODS

### Study Design and Patient Selection

This study was approved by the hospital system’s institutional review board. The two vendors used in this study entered into a master security agreement with the institution, and both entered a business associate agreement to maintain private health information and ensure Health Insurance Portability and Accountability Act of 1996 compliance. This was a retrospective, qualitative study conducted at a not-for-profit healthcare system with nine acute care hospitals in the Mid-Atlantic Region, including tertiary-care, urban academic hospitals and rural community hospitals, with a combined annual ED volume of 430,000 visits. Any ED patient who was diagnosed with COVID-19 between December 2020–August 2021 was considered for monitoring. Qualifying patients were identified by their treating physician, physician assistant, or nurse. Patients were offered enrollment 24 hours a day, and the enrollment population consisted of patients residing in both metropolitan and rural geographies. Clinicians were instructed to be insurance agnostic; all patients were eligible regardless of insurer or insurance status.

Criteria for inclusion in the RPM program were as follows:

• Patient in the ED had new diagnosis of COVID-19 within the prior seven days
• Disposition from ED visit was characterized as “discharge to home”
• Patient consented to monitoring (or parent/legal guardian consented if the patient was <18 years old)
• Patient had reliable access to a mobile phone or land line (did not need to be the patient’s own phone; could belong to family member or friend)
• Patient interested in program enrollment
• Clinician discretion (They were encouraged but not required to enroll all eligible patients)

Criterion for exclusion:

• Patient not interested in, or not consenting to, monitoring

Two forms of consent were obtained: verbal consent (clinician discussed the program with the patient and determined patient interest) and written consent (embedded within the RPM mobile app, prior to initialization). For patients <18 years old, the parent or legal guardian consented to and operated the app and transmitted data on behalf of their child. No patient under the age of 18 handled the device independently.

Patients received a kit containing a pulse oximeter and thermometer. Once a patient consented to monitoring, the clinician placed an order for “COVID Home Monitoring” in the electronic health record (EHR), which assigned the patient an RPM enrollment ID number (matched to the ID number on the pulse oximeter). On the RPM platform, patient identifiers were removed, and patients were referred to by enrollment ID number only. If needed, the RPM clinician could reference a secure report of all patients who had received a “COVID Home Monitoring” order (generated within the EHR) to match a patient’s enrollment ID number back to their medical record number.

Two RPM platforms were used for this program, which we will refer to as Platform 1 and Platform 2. The two platforms differed in design. Active monitoring on Platform 1 was introduced in December 2020; patients were asked to self-report oxygen saturation and symptomatology on the platform using the mobile app. Platform 2 was introduced in March 2021 to simplify the data collection process: its Bluetooth-integrated pulse oximeter automatically uploaded oxygen saturation and heart rate data to Platform 2 as soon as the pulse oximeter was applied to the patient’s finger, eliminating the need for manual entry. Patients without smartphones were issued a traditional (non-Bluetooth) pulse oximeter and vital sign data was solicited and entered manually by the monitoring team. Patients who met inclusion criteria but did not enroll, or patients who enrolled but did not submit any data, were considered the non-RPM group.

The RPM team was comprised of nurse practitioners and medical assistants, with coverage seven days a week from 9 am to 5 pm. Patients were monitored for a maximum of 16 days, with the option to disenroll at any time during that period. During that time, patients uploaded vital signs as frequently as desired. Any patients with ongoing COVID-19 symptoms after 16 days (but without vital sign abnormalities) were referred to our institution’s COVID-19 Recovery Program. Participants kept the pulse oximeter after completion of the program.

Participation was insurance-agnostic and free of charge to the patient. A subset of patients identified a primary language other than English; for these, we used interpreter services during interactions. The default language on both RPM apps could be toggled to English or Spanish.

### Interventions and Measurements

Oxygen saturation and heart rate data were collected each time the patient applied the pulse oximeter. With each check-in, patients also had the option of reporting temperature and symptoms. Alert parameters were embedded within the digital platform; the RPM team received an alert when a concerning symptom (ie, chest pain or dyspnea) or vital sign reading (ie, SpO_2_ < 94% or heart rate >100) was submitted, and contacted the patient via video call, phone call, or in-app message. If appropriate, patients were referred back to the ED. We encountered several spurious SpO_2_ readings due to poor contact between the pulse oximeter and the patient’s finger; these values improved after adjustment of pulse oximeter placement with coaching from the RPM clinician.

Alerts received after hours triggered an automatic reply advising ED precautions. The RPM care team followed up with these patients the next day. Alerts were set for “missed vitals”—several days without data transmission—which prompted a call from the RPM team. Any technologic difficulties were addressed with troubleshooting via phone call from the RPM team. Throughout the monitoring period, patients could initiate communication with the RPM team at any time via the chat function or by calling the RPM support number.

### Outcomes and Analysis

Our primary outcome was a descriptive analysis of patients enrolled in the COVID RPM program (including total number of patients, patient age, patient gender, racial distribution, their engagement (number of SpO_2_ readings uploaded per patient, average SpO_2_ reading, and number of days of engagement per patient). Our secondary outcomes were descriptions of 30-day returns to ED, number of days between discharge and return visit, ED diagnosis at return visit, and disposition from return visit for the RPM vs non-RPM group, using an as-treated analysis.

## RESULTS

Population characteristics are noted in Table 1. The RPM group was comprised of younger patients (median age of 47 years vs 52 in non-RPM group) and a higher percentage of female patients (61.1% female vs 55.4% in the non-RPM group). There was a slight predominance of patients identifying as Black in the RPM group (64.4% vs 60.6% in the non-RPM group). Fewer RPM patients were enrolled in Medicaid (21% vs 35% in non-RPM group) and more enrolled in a managed care plan (25% vs 22% in the non-RPM group). Gender was extracted from the EHR, and there were no participants who identified as non-binary.

**Table 1.** Study population characteristics.

	RPM	Non-RPM
Median age in years (IQR)	47 (23)	52 (32)
Female gender	61.1%	55.4%
Male gender	38.9%	44.6%
Racial distribution		
Black	64.4%	60.6%
White	21.0%	28.1%
Other	14.6%	11.3%
Insurance at emergency department visit		
Unknown	36%	4%
Medicaid	21%	35%
Managed care	25%	22%
Medicare	10%	24%
Self-pay	6%	10%
Commercial	1%	4%

*RPM*, remote patient monitoring; *IQR*, interquartile range.

Overall, 52.2% of patients with a new diagnosis of COVID-19 were discharged home, of whom 41.4% were enrolled on RPM (Table 2). The remaining patients were predominantly placed in inpatient admission, followed by observation and transfer. Of those enrolled, 51.4% were active and engaged, reporting at least one vital sign or symptom during the monitoring period.

**Table 2.** Remote patient monitoring enrollment following COVID-19 emergency department visits.

	Patient volume	% of Total
Total COVID+ ED visits 12/1/2020–8/31/2021	16,013 (14,127 unique patients)	
Discharged to home	8,357	52.2%
Enrolled on RPM	3,457	41.4%
Active on RPM^1^	1,779	51.4%
Non-RPM^2^	6,578	78.7%
Admission	5,749	35.9%
Observation	1,101	6.9%
Other (transfer, discharge to rehabilitation or skilled nursing facility, elopement, against medical advice, or deceased)	806	5.0%

^1^ Reported at least one vital sign or symptom during the monitoring period.

^2^ Met inclusion criteria but not enrolled or referred but not active.

*COVID-19*, coronavirus 2019; *ED*, emergency department; *RPM*, remote patient monitoring.

**Figure 1.**
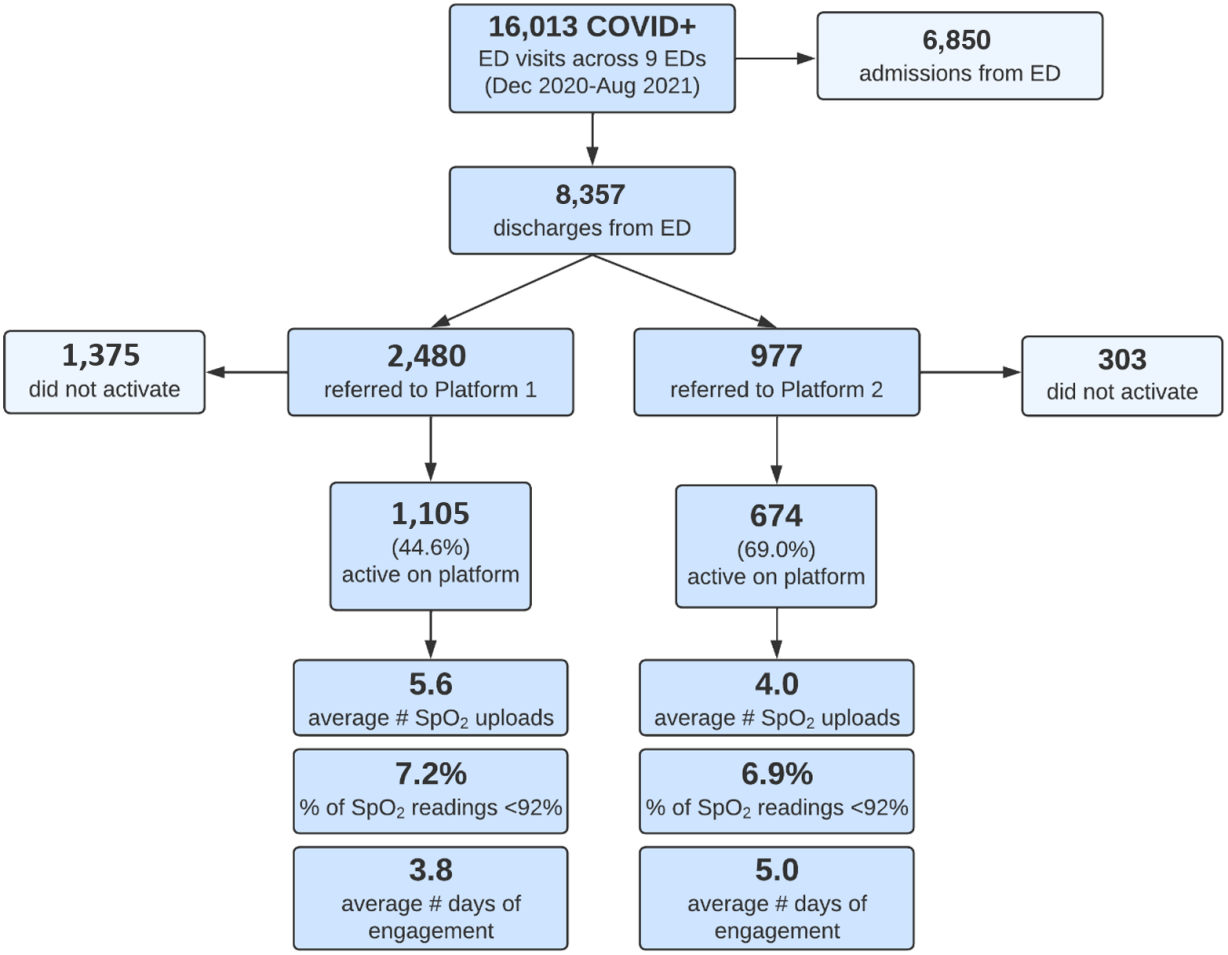
Study size and engagement by platform. *COVID-19*, coronavirus 2019; *ED*, emergency department; *SpO_2_*, oxygen saturation.

Further examination of the active patient population revealed differing engagement between platforms (Figure 1). The Bluetooth-enabled platform demonstrated a higher percentage of active patients (uploading at least one point of data) and longer duration of engagement (5 days vs 3.8 days). Patients using the non-Bluetooth-enabled platform uploaded more points of data on average. The percentage of patients with SpO_2_ reading < 92% was similar on both platforms.

In the RPM group, we observed a lower rate of 30-day return to ED compared to the non-RPM group (Table 3). Mean number of days between discharge and return and mean number of return-to-ED episodes within 30 days were similar. Of 30-day returns to the ED, a higher percentage of patients in the RPM group required admission or observation at the second visit (47.7% vs 34.8%). Most 30-day returns were coded with diagnoses of sepsis, chest pain, urinary tract infection, and pulmonary embolism. The non-RPM group had a higher percentage of patients diagnosed with viral diseases complicating third trimester pregnancy.

**Table 3.** 30-day returns for patients with COVID-19 diagnosis.

	RPM	Non-RPM
Patients with 30-day return to ED	111/1779 (6.2%)	980/6578 (14.9%)
Mean days between discharge and return	5.1 ± 4.4	5.2 ± 4.6
Mean return-to-ED episodes within 30-day period	1.0 ± 0.2	1.1 ± 0.4
Disposition of 30-day return-to-ED visit		
Discharge to home	55 (49.5%)	581 (59.3%)
Admission or observation	53 (47.7%)	341 (34.8%)
Other	3 (2.7%)	58 (5.9%)
Diagnosis for 30-day ED returns		
COVID-19	91	723
Other specified sepsis	9	43
Other viral diseases complicating pregnancy, third trimester	0	6
Other chest pain	2	5
Urinary tract infection, site not specified	0	5
Other pulmonary embolism without acute cor pulmonale	0	5
Contact with and (suspected) exposure to COVID-19	0	4
Bacteremia	0	3
Unspecified abdominal pain	0	3
Other viral diseases complicating pregnancy, first trimester	0	3
Anxiety disorder, unspecified	1	0
Other viral diseases complicating pregnancy, first trimester	1	0
Cerebral edema	1	0
Other fatigue	1	0
Type 2 diabetes mellitus with hyperglycemia	1	0
Single subsegmental pulmonary embolism without acute cor pulmonale	1	0
Hypertensive chronic kidney disease with stage 5 chronic kidney disease or end stage renal disease	1	0
Calculus of gallbladder and bile duct with acute cholecystitis without obstruction	1	0
Other	0	180

*COVID-19*, coronavirus 2019; *ED*, emergency department; *RPM*, remote patient monitoring.

**Figure 2.**
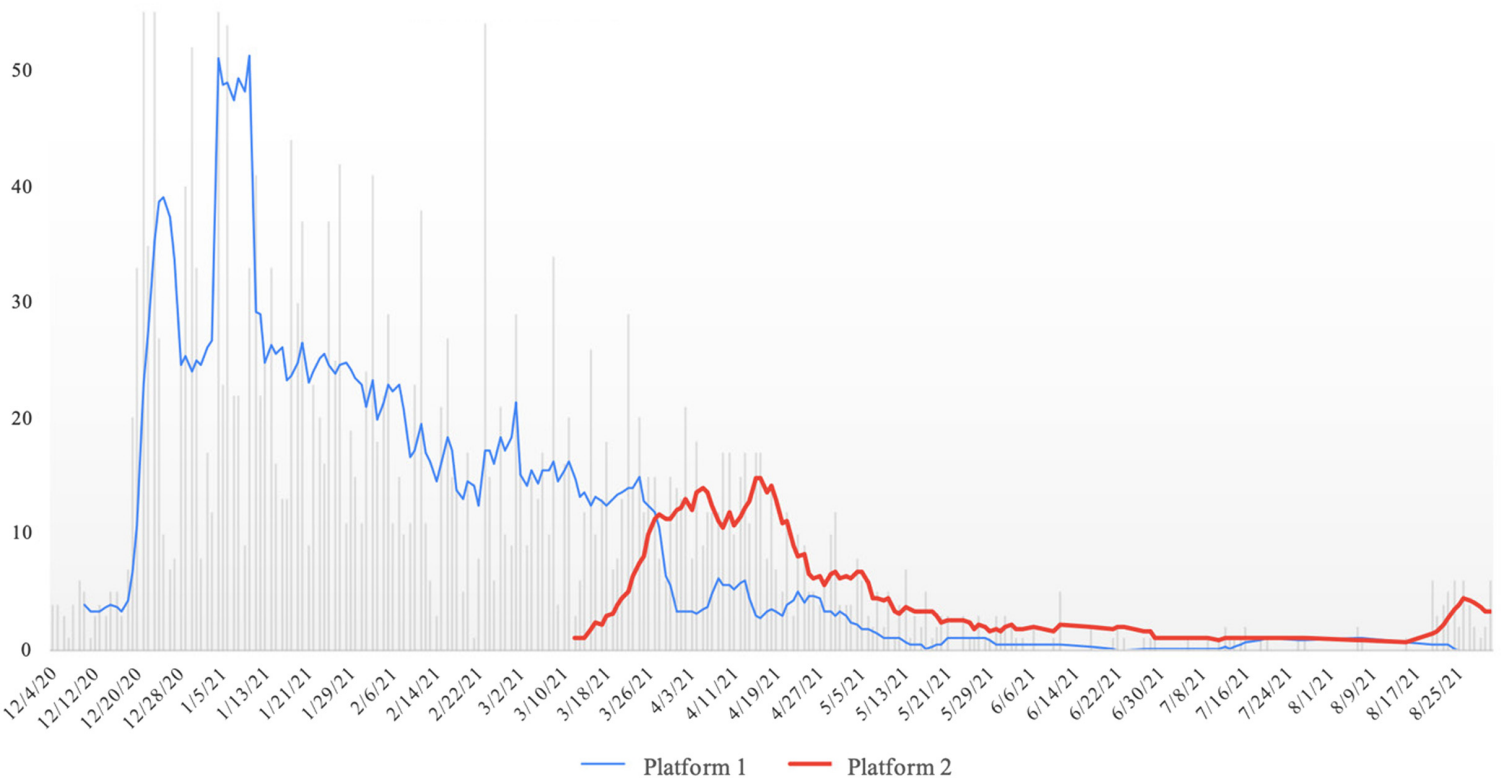
Remote patient monitoring referrals, 7-day rolling average, December 2020–August 2021.

**Figure 3.**
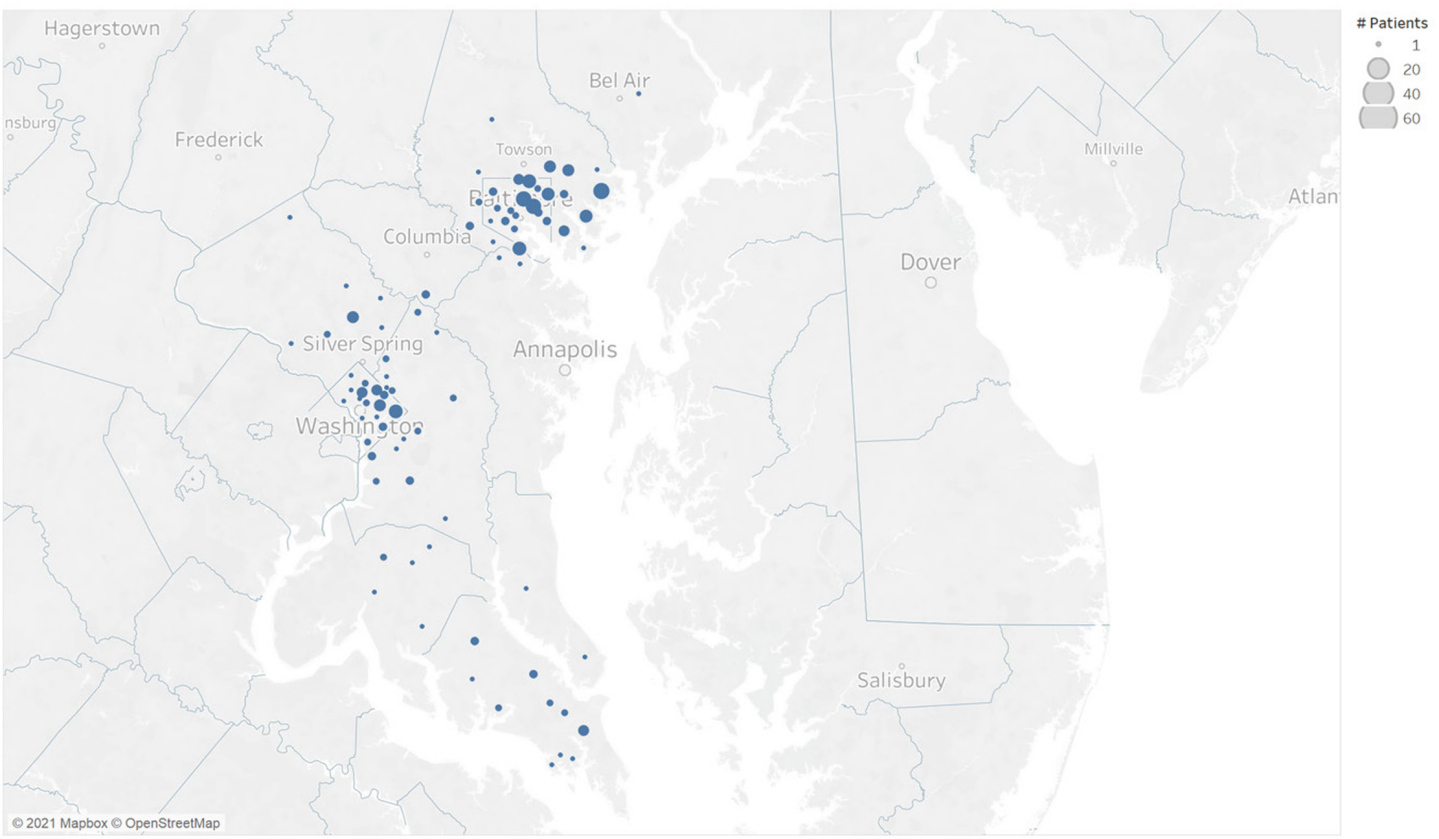
Geographic distribution of patient population by ZIP Code.

RPM referrals peaked in December 2020 and January 2021 (Figure 2). In March 2021, we adopted a Bluetooth-enabled device (Figure 2, Platform 2) and phased out the non-Bluetooth device. Geographically, participants were distributed widely across the metropolitan Washington, DC, and Baltimore areas as well as surrounding rural regions (Figure 3). The RPM activity was not limited to geographies adjacent to hospitals where enrollment took place.

## DISCUSSION

RPM expands the scope of available “clinical space” beyond the brick-and-mortar constraints of the hospital, which was especially useful given rapid variation in inpatient capacity during the pandemic surge. Given the highly individual trajectory of COVID-19 disease progression, patients can remain stable for several days before decompensation;^6^ it may be in the patient’s best interest to recover at home during this latent period, due to risk of nosocomial infection, deconditioning, and financial and social burden associated with hospital admission.^7^ For patients with deteriorating clinical status, physiologic monitoring prompts them to return to the ED sooner than with symptomatic monitoring only,^8^ due to higher sensitivity of oxygen saturation reading compared to self-reported dyspnea.^9^ In particular, patients with COVID-19 often experience “silent hypoxemia”^10^—hypoxemia in the absence of symptoms—which can lead to delayed care.

Of the 8,357 COVID-19 positive patients in our study who met inclusion criteria, 3,457 were referred to RPM, and 1,779 enrolled onto the platform. Our enrollment of 1,779 patients made this one of the largest published COVID-19 RPM programs to date with physiologic monitoring, a demonstration of an innovative post-acute digital patient engagement at multihospital system scale. A COVID-19 RPM program at Kaiser Permanente Southern California, which also monitored a large population of patients,^11^ had two primary differences: insurance status of participants and the use of Bluetooth-enabled devices. Kaiser enrolled patients from within its insurance program, while our study enrolled all comers regardless of insurance status, including patients with Medicaid or who were uninsured. Our use of Bluetooth-enabled devices led to increased engagement on the platform (69.0% vs 44.6% engagement on the Bluetooth vs non-Bluetooth-enabled platform), presumably due to a simplified process for uploading vital signs. However, our overall compliance was lower compared to Kaiser (51% vs 94%). This may be due to Kaiser’s more thorough patient education at the point of enrollment.

Our study is one of the few to examine both physiologic and symptomatic data in an all-comer ED population, agnostic to insurance.^11^ ^–^ ^17^ In addition, we built workflows to accommodate patients who have historically been excluded from RPM: patients without smartphones or with limited English proficiency.^7^ ^,^ ^18^ ^–^ ^20^ This was done to decrease a selection bias commonly seen in technology-based interventions. Of 1,779 enrollees, 120 did not own a smartphone. For these patients, the RPM team dedicated time to calling patients daily, with the goal of providing the same level of monitoring and virtual care that was available to patients with smartphones. For this population, RPM nurse practitioners manually recorded patient-reported physiologic and symptom data, in lieu of automatic data upload from Bluetooth-enabled pulse oximeters. We additionally encouraged the use of RPM via surrogate (ie, recruiting a family member, friend, or home health aide to input data on behalf of a patient who was not facile with the digital platform). Forty-three patients spoke a primary language other than English (including Spanish, Portuguese, and Thai). Both platforms were made available in Spanish; all other non-English interactions were undertaken with the assistance of an interpreter service. We acknowledge that despite a dedicated effort to lessen these disparities, we did not fully eliminate them. Bias that persisted was due in part to clinician discretion in patient enrollment.

Of note, the median age of those in the RPM program (47 years old) was lower than their non-RPM counterpart (52 years old). This may be due to clinician enrollment bias, higher likelihood of admission or observation on index ED visit for older patients (rather than discharge to home), or higher technologic fluency among younger patients. Both the RPM and non-RPM groups included a greater percentage of patients identifying as female as opposed to male.

We observed a lower 30-day return-to-ED rate for patients on RPM (6.2%) compared to patients not on RPM (14.9%), and the average number of return-to-ED episodes within that 30-day period was lower for RPM (1.0 ± 0.2) than for non-RPM (1.1 ± 0.4). Additionally, we observed a higher rate of admission or observation at return ED visit (47.7%) for the RPM group (47.7% vs 34.8%).

Compared to the non-RPM cohort, we saw greater enrollment from patients insured by Blue Cross Blue Shield. While the RPM cohort was smaller than the non-RPM cohort, which could have led to distortion of payer distribution, we must also consider the effect of enrollment bias. Insurance status (specifically Medicaid) is commonly used as a proxy for socioeconomic status. Although we aimed to enroll patients regardless of insurance status, it is possible that other factors dissuaded against RPM enrollment, including the lack of reliable access to a phone number and Wi-Fi or data plan, technologic fluency, or housing. While unintended, these biases affect healthcare delivery. Future RPM interventions should implement an enrollment process that identifies and counteracts any categorical barriers posed by technical or medical literacy, access to Wi-Fi or data, and age.

Finally, we learned lessons in supply management. We instated a Cerner order for “Referral to COVID-19 Home Monitoring”; this was essential for tracking device distribution at each ED site. Our supply management was overseen centrally; a logistics coordinator contacted each ED site monthly to determine device supply needs. In designing the program, we opted for an entirely virtual care team, which allowed for easy scalability in work force and more agility in staffing in response to epidemiologic trends. Remote staffing of one nurse practitioner and one medical assistant was sufficient to monitor all enrollees.

There is ample opportunity for future research. Interesting questions include the following: Are participants less likely to return to the ED for non-urgent COVID-19 symptoms, with the knowledge that their SpO_2_ was in the acceptable range? Does immediate access to a virtual clinical team change a patient’s return-to-ED behavior? Does the presence of an RPM program change clinician behavior in deciding discharge vs admission? For participants who did not stay enrolled for the full duration of monitoring, what factors led to disenrollment? All these questions bear further investigation.

The COVID-19 RPM program was retired in April 2022. In the interim between this study period and completion of the program, we expanded enrollment to include patients from our outpatient monoclonal antibody infusion clinics and select inpatient COVID-19 units. In total, we referred 6,294 patients to RPM and enrolled 2,937. While the focus of this study was RPM in the context of COVID-19, we can apply lessons learned and workflows to other acute disease states (eg, pneumonia, acute decompensated heart failure, chronic obstructive pulmonary disease exacerbation). From February–April 2022, we implemented a parallel ED RPM program for patients with pneumonia and non-COVID-19 respiratory viral illness, using the same clinical protocols. We have received overwhelmingly positive responses from patients and their families, as well as from clinicians.

## LIMITATIONS

This study has several limitations. First, as a non-matched retrospective observational study, we cannot conclude that any difference in return-to-ED or readmission rate was attributable to the use or non-use of RPM. Patient enrollment was not blinded, and not all patients who qualified for RPM were enrolled, thus creating opportunity for bias in enrollment. Variables that introduce bias include the following: patient selection (individual clinician determines which patients would benefit most from RPM); shift mechanics (clinicians are less likely to enroll a patient during a particularly busy shift); and patient’s preferred language (patients with limited English proficiency may be less likely to be enrolled due to the additional step of using an interpreter service.) Another limitation due to our study design was information bias; some patients sought additional care at a different medical system and thus were lost to our 30-day return-to-ED data collection. Therefore, we were not able to fully account for the outcomes of all patients.

There were also external confounding factors. With rapid shifts in ED practice patterns (threshold determination of which patients are safe for discharge), hospital inpatient capacity, and community prevalence of COVID-19, the number of patients who were discharged and enrolled on RPM varied widely during our study period. Therefore, the population characteristics of both the RPM-enrolled and non-enrolled groups varied. During our nine-month enrollment period, we acknowledged the need to iterate on both hardware and workflow in response to rapidly changing COVID-19 and RPM landscapes. For example, the increasing prevalence of Bluetooth-enabled pulse oximetry led to our pivot from manual data entry to automated Bluetooth-enabled data upload. Consequently, patients were offered one of two different RPM pulse oximeter devices and platforms depending on their time of enrollment.

We recognize as well that using an as-treated analysis may overestimate the difference in 30-day return-to-ED calculations. For example, patients who do not engage with the platform and pulse oximeter may also be more likely to return to the ED due to underlying medical or social factors. However, many patients who were “enrolled but not active” did not receive a pulse oximeter kit or download the app. In these cases, the emergency physician placed a “Refer to RPM” EHR order, but the patient did not have the opportunity to use the pulse oximeter or communicate with our team.

Finally, while we were able to capture the majority of patients who were referred to our program, a subset (1,678 of 3,457 patients referred) did not successfully connect to the platform. This was due to several reasons, including lack of pulse oximeter distribution or app download at discharge; technologic difficulty (with either the pulse oximeter or app); lack of consistent phone access; or loss of patient interest. Patients with higher technologic and medical fluency were more facile with operating the app, which contributed to self-selection bias in enrollment. While outside the scope of this study, further analysis of causes for lack of patient engagement would be valuable.

## CONCLUSION

Remote patient monitoring is a versatile tool to expand our scope of care delivery. There is a paucity of data on the long-term significance of at-home monitoring, especially as it relates to engagement with care and return-to-ED patterns in patients commonly excluded from RPM. This study does not imply causation and may not apply broadly, due to differences in study population. However, it contributes to our current knowledge of large-scale RPM implementation and can be used as a building block to continue exploring the functionalities and clinical strengths of RPM.
